# Fully-Textile, Wearable Chipless Tags for Identification and Tracking Applications †

**DOI:** 10.3390/s20020429

**Published:** 2020-01-12

**Authors:** Laura Corchia, Giuseppina Monti, Egidio De Benedetto, Andrea Cataldo, Leopoldo Angrisani, Pasquale Arpaia, Luciano Tarricone

**Affiliations:** 1 Department of Engineering for Innovation, Complesso Ecotekne-Corpo O, University of Salento, 73100 Lecce, Italy; laura.corchia@unisalento.it (L.C.); giuseppina.monti@unisalento.it (G.M.); andrea.cataldo@unisalento.it (A.C.); luciano.tarricone@unisalento.it (L.T.); 2 Department of Information Technology and Electrical Engineering, University of Naples Federico II, 80125 Naples, Italy; angrisan@unina.it (L.A.); pasquale.arpaia@unina.it (P.A.)

**Keywords:** chipless tag, frequency shift, identification, IoT, microwave resonators, textile, tracking, wearable

## Abstract

In this work, two fully-textile wearable devices, to be used as chipless identification tags in identification and tracking applications are presented. For the fabrication of the fully-textile tags, a layer of fleece was used as a substrate, while an adhesive non-woven conductive fabric was employed for the conductive parts. To allow radio-frequency identification of these chipless tags, two alternative techniques were used. One relies on associating a binary code with the resonance frequency of resonant devices: the presence/absence of the resonance peaks in the transmission scattering parameter, $|S_{21}|$, of a set of resonators is used to encode a string of bits. The second technique for accomplishing radio-frequency identification of the chipless tags resorts to a frequency-shift coding technique, which is implemented by modifying the configuration of a hairpin resonator. The obtained numerical and experimental results confirm the suitability of the proposed strategies for obtaining entirely-textile, wearable chipless tags for identification and tracking purposes, which can be particularly useful, especially in the industrial sector. In this field, in fact, the proposed solutions would guarantee a seamless integration with clothes and would facilitate the user’s interaction with the IoT infrastructure. In this regard, one of the envisaged application scenarios related to the tracking of hides in the leather industry is also presented.

## 1. Introduction

The Internet of Things (IoT) relies on smart environments, equipped with wireless sensor networks [1] and synergistic personal area networks [2,3], in which the user must be uniquely identified, while he/she interacts with the IoT infrastructure. One challenge, however, still remains the need to facilitate a seamless integration of IoT solutions with the surrounding context [4,5,6], and especially with people. Because clothing can be adapted to everyday life, wearables (combined with smart textiles) are arguably the best candidates to facilitate this process, possibly becoming the next interface between the real and the digital world [7]. As a result, the development of wearables that could allow to effectively engage the user into the IoT sensory environment without compromising their comfort has become a major interest of applied research [8,9,10]. Nevertheless, despite its potential, the use of wearable electronics has been hindered by some practical limitations, such as its robustness to basic but severe operations (e.g., washing, ironing) [11,12] and the need to tin solder the necessary integrated circuitry (IC). Even the European Commission has recently stated that future wearables will have to be “shapeable, stretchable and washable/cleanable on-demand”, emphasizing that a wearable should look like natural clothing in terms of comfortability, breathability and washability [13]. One of the strategies to overcome these limitations is to use textile materials for fabricating the devices [14,15] and to resort to chipless devices [16]. This would lead to a potentially infinite service-life of the devices, and it would also lower energy consumption. In an industrial IoT sensory environment, the proposed strategies could be implemented to work, for example, as chipless identification tags or, in general, as wearable sensors [17,18,19,20,21,22,23].

Starting from these considerations, in this work, the design, fabrication and characterization of fully-textile, chipless tags for wearable applications are presented. This objective is accomplished by implementing two strategies. The first is the combined use of a layer of fleece (a synthetic fabric commonly used in clothing industry) as a substrate and of an adhesive non-woven conductive fabric for the conductive parts: this allows to obtain fully-textile devices. The second strategy relates to the adoption of encoding/decoding technique that relies on the “frequency signature” of the textile device, and which allows to avoid the use of on-board electronics. The final result is to obtain entirely textile identification tags, able to guarantee a seamless integration with clothes.

It is important to point out that, in the literature, only two chipless tags for wearable applications have been proposed [24,25]. One was a chipless tag consisting of three scatterers sewn on a cotton substrate [24]: this device worked well in free space, but its performance degraded when placed in proximity to the human body (closer than 7.5 mm). While in [25], a chipless tag consisting of multiple L-shaped scatterers was investigated. This chipless tag could depolarize the incident wave and create a response in the orthogonal polarization; however, the experimental results reported in [25] refer to prototypes fabricated on a rigid substrate commonly used for PCB, and the extension of these results to fully-textile materials is not straightforward [15,26].

The present paper is organized as follows. In Section 2, the design and fabrication of two fully-textile identification tags are addressed. The experimental characterization of the two prototypes is reported in Section 3. Successively, in Section 4, the effect of bending and the effect of the human body on the performance of the proposed devices are addressed. In Section 5, one of the envisaged applications of for tracking purposes in the leather manufacturing industry is presented. Finally, in Section 6, conclusions are drawn. With respect to previous works, in this paper additional numerical and experimental results have been added. In particular, for the three-resonator device, the simulation results of all the eight configurations are presented. For the hairpin resonator structure, the experimental tests for the different configurations are included. Moreover, the bending effect and the influence of the human body on the performance of the devices are addressed. Finally, the application case on leather has been introduced.

## 2. Design and Fabrication of Two Fully-Textile Tags

Two fully-textile chipless tags are presented, each employing a different encoding strategy for accomplishing radio-frequency identification without electronic chips. One solution is to employ a multi-resonator device and to consider its transmission scattering parameter ($S_{21}$): the presence/suppression of the resonant behaviour of each single resonator could be associated to a logic ‘1’ or ‘0’ (resonance-based coding). Another alternative, investigated with the second structure, is to resort to a frequency-shift coding technique. In this case, the geometry of a resonator is modified to shift its resonance frequency ($f_{res}$): hence, each $f_{res}$ value can be associated, for example, to a string of bit or to an identification code.

### 2.1. Materials

To guarantee integration with clothes, only fully-textile materials were considered. A 1 mm-thick bi-layer of fleece was used as a substrate. For designing the prototypes, it was necessary to assess the value of the relative dielectric permittivity of fleece, $\varepsilon_{r,f}$, to be used in the full-wave simulations. To this purpose, an iterative procedure was carried out. In particular, several rectangular patch antennas using the fleece as a substrate were designed (assuming tentative values for $\varepsilon_{r,f}$), fabricated and characterized. The differences between the expected performances and the actual ones were used to determine, iteratively, the optimal value of $\varepsilon_{r,f}$ to be used for the successive full-wave simulations. The obtained value for $\varepsilon_{r,f}$ was 1.18.

For the conductive parts, a 0.11 m-thick non-woven conductive fabric (NWCF) was used (electrical conductivity approximately equal to 2.27 × 10${}^{5}$ S/m). This NWCF, which is made of nickel and copper, is manufactured by Saint Gobain company [27].

### 2.2. Fully-Textile Tag Relying on Resonance-Based Coding

**Figure 1:**
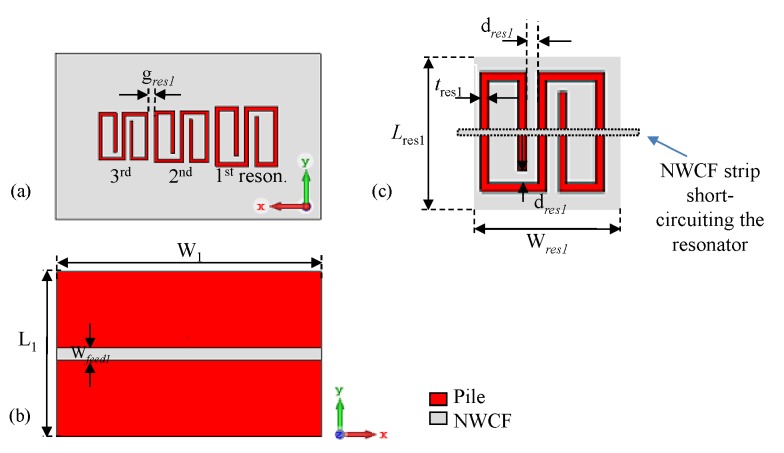
Design of the multi-stopband structure: (**a**) resonators on the back; (**b**) microstrip line on the front; (**c**) zoom of one resonator with details on how the resonance suppression was obtained. *©*[2019] IEEE. Reprinted, with permission, from [19].

The idea at the basis of resonance-based coding is to associate the presence of the resonance in the $S_{21}$ of a microwave resonator, for example, to a logic ‘1’. Likewise, the suppression (or absence) of the resonance could be associated to a logic ‘0’. In this way, a multi-resonator structure with *n* resonators, would allow to have as many possible bits available [16,28]. To demonstrate the feasibility of this strategy, a multi-stopband structure consisting of a 50 Ω microstrip line loaded by three resonators was designed, fabricated and characterized. The geometry and dimensions of the structure were optimized through full-wave simulations (carried out in CST Microwave Studio). Figure 1 shows a sketch of the three-resonator structure; it can be seen that the resonators are slotted in the ground plane of the microstrip line. The three resonators were designed so that each one would resonate at a different frequency ($f_{res1}$ = 2.1 GHz; $f_{res2}$ = 2.4 GHz; and $f_{res3}$ = 2.7 GHz).

**Figure 2:**
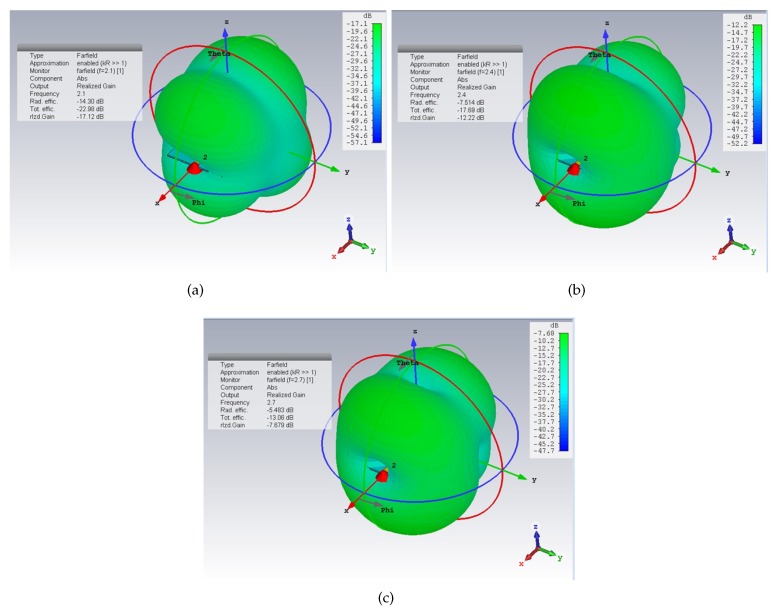
Far-field radiation patterns for the multi-stopband device: at 2.1 GHz (**a**); at 2.4 GHz (**b**); and at 2.7 GHz (**c**).

To analyze possible radiating effects caused by the slots in the ground plane, the far-field and the near-field behaviour of the proposed devices were analyzed through full-wave simulations. Figure 2 shows the results obtained for the far-field radiation patterns. In particular, for each resonance frequency value, the far-field radiation pattern of the device with the three slotted resonators was considered. It can be noticed that the radiated energy is negligible.

**Figure 3:**
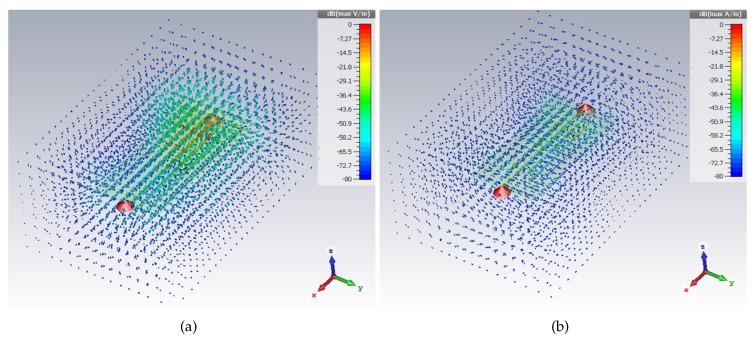
Simulation results of the electric field (**a**) and of the magnetic field (**b**), evaluated at 2.1 GHz, for the multi-stopband device.

As for the near-field effects, full-wave simulations were carried out to analyze the behavior of the electric and magnetic fields. Figure 3a,b show the electric and magnetic fields, respectively, calculated at 2.1 GHz. It can be noticed that both fields decrease quickly with the distance.

With regard to the design frequency, it is worth mentioning that, at the state of the art, there is not a standard that regulates the operating frequency of chipless tags. Some works have employed devices working in the 3.1–10.3 GHz frequency band [29,30]. However, because the facilities in the availability of the authors were not suitable for fabricating devices in this frequency range, it was decided to design and fabricate tags with resonators working at lower frequencies, although close to the ones addressed in the literature. After optimizing the geometry and the layout of the first resonator (so that it would work at $f_{res1}$), the dimensions were scaled so that the second and third resonators would resonate at $f_{res2}$ and $f_{res3}$, respectively. The resulting dimensions are reported in Table 1.

**Figure 4:**
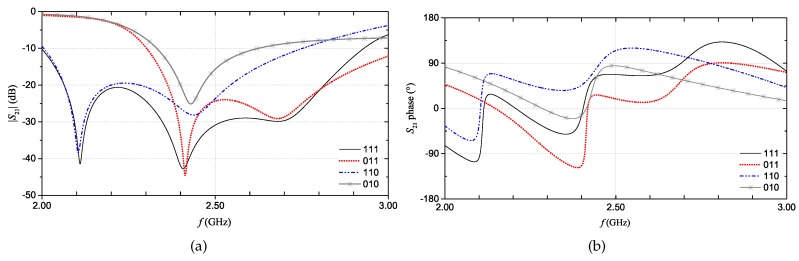
Multi-stopband structure. $S_{21}$ simulations results for four coding configurations: magnitude (**a**) and phase (**b**). *©*[2019] IEEE. Reprinted, with permission, from [19].

The full-wave simulation results for the reflection scattering parameter, $S_{21}$, corresponding to four configurations of the multi-stopband structure are shown in Figure 4. For the sake of example, let us consider the $|S_{21}|$ curve where three resonant peaks are simultaneously present; this curve can be associated to the ‘111’ code. On the other hand, the $|S_{21}|$ curve in which only the resonant peak at approximately 2.4 GHz is present (i.e., the resonance of the second resonator) can be associated to the ‘010’ code. Similar considerations apply for the other $|S_{21}|$ curves. Basically, by considering the $S_{21}$ of the chipless device, it is possible to extrapolate the associated binary code. As can be observed from Figure 4b, also the $S_{21}$ phase changes depending on the presence/absence of the resonances.

**Figure 5:**
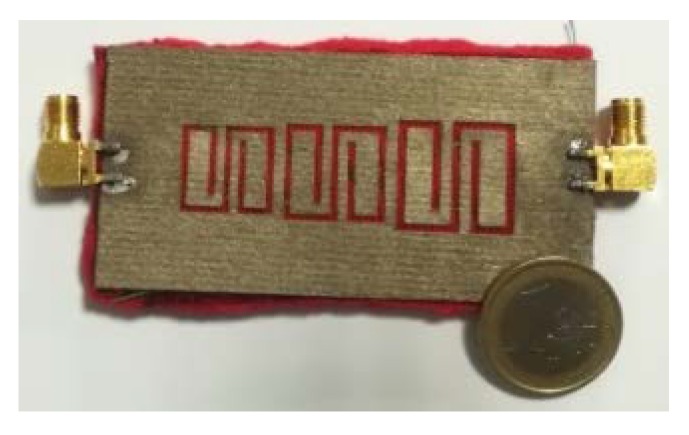
Picture of the multi-stopband structure. *©*[2019] IEEE. Reprinted, with permission, from [19].

Figure 5 shows a picture of the back of the multi-stopband prototype device. The NWCF was shaped by using a cutting plotter (model CraftROBO CE5000-40 disributed by Graphtec). The adoption of NWCFs allows to reduce the manufacturing costs in comparison to solutions based on electro-textiles or conductive threads [24,25,26]. In fact, NWCFs are generally less expensive and, most importantly, they do not fray.

### 2.3. Fully-Textile Tag Relying on Frequency-Shift Coding

**Figure 6:**
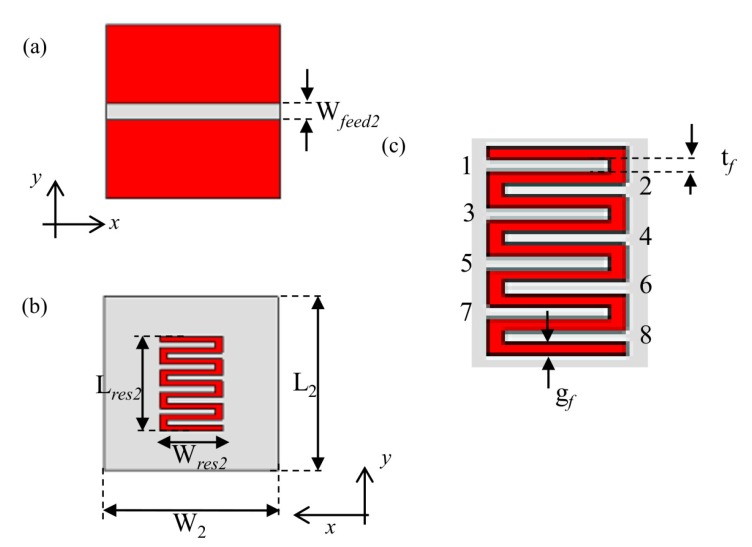
Geometry and design parameters of the proposed fully-textile interdigital hairpin resonator: (**a**) front view; (**b**) back view; (**c**) geometrical parameters of the fingers. *©*[2019] IEEE. Reprinted, with permission, from [19].

The second encoding technique is based on frequency shift. Figure 6 shows the layout of the proposed resonant structure, which consists of a 50 Ω microstrip line loaded with an interdigital hairpin resonator (with eight fingers). Also in this case, to reduce the overall size of the structure, the resonator is slotted on the ground plane of the microstrip line. Indeed, a chipless tag based on interdigital hairpin resonant structures was proposed in the literature [31]. However, in [31], each resonator was used to encode a single bit. On the other hand, in the proposed prototype, the data encoding is achieved through a selective removal of one or more fingers of the hairpin structure. Selectively cutting out the fingers leads to variation of the resonance frequency: hence, each configuration of the resonator may be associated to a different code.

The resonance frequency can be varied within a pre-established frequency band (FB), which can be used as a design parameter. In turn, FB can be divided into different sub-bands, which can be referred to as resolution bands (Δf): when the resonance frequency falls within a pre-established Δf range, this can be associated to a specific identification code. The design values of FB and of Δf should be decided considering the following aspects:

- the dimensional resolution achievable with the available manufacturing process; and
- the accuracy and frequency resolution of the measurement setup used to characterize the device [32].

Often, the first aspect is the most stringent, especially when fabricating complex geometries.

On the basis of these considerations, for the prototype, the range 1.80–3.20 GHz was considered as FB, and the width of each resolution band was chosen approximately equal to Δf = 140 MHz. Table 2 summarizes the dimensions of the hairpin resonator, after optimization. Also in this case, the design was carried out through CST Microwave Studio.

**Figure 7:**
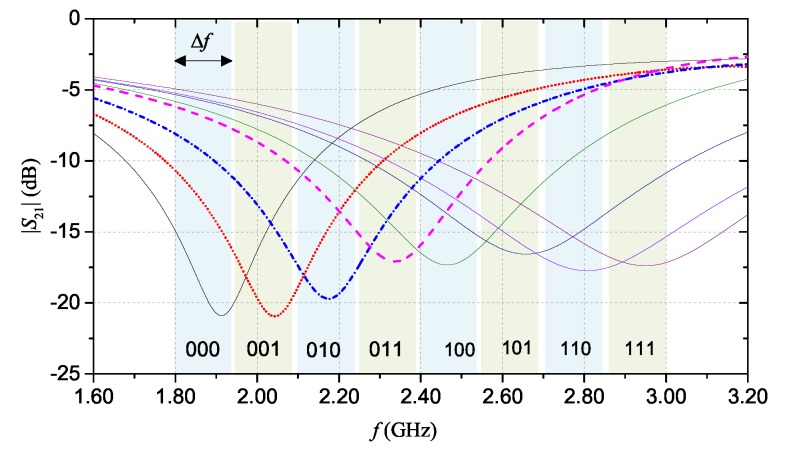
$|S_{21}|$ simulation results for the hairpin resonator. Each curve corresponds to a different configuration of the resonator. *©*[2019] IEEE. Reprinted, with permission, from [19].

**Figure 8:**
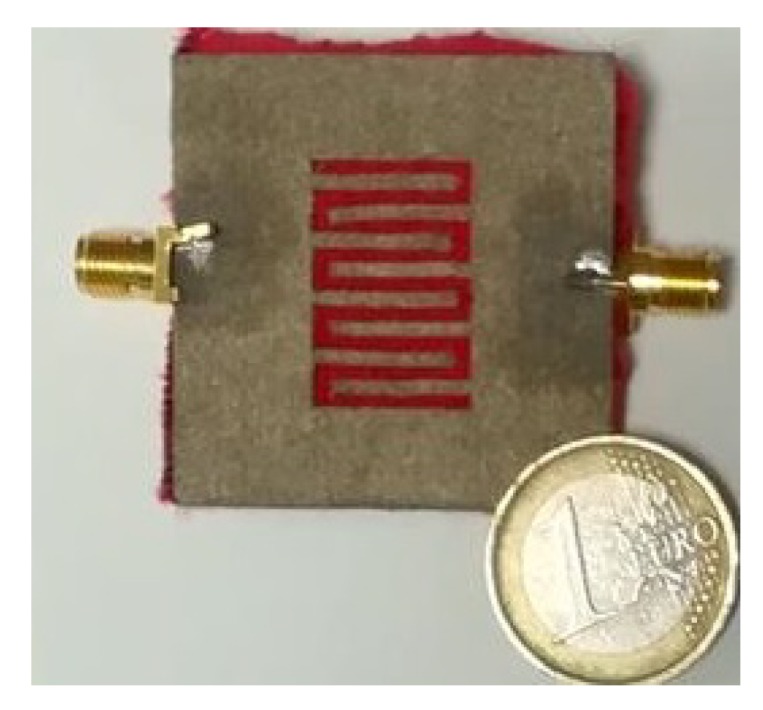
Picture of the back of the hairpin resonator. *©*[2019] IEEE. Reprinted, with permission, from [19].

Figure 7 shows the results of the full-wave simulations. As aforementioned, each $|S_{21}|$ curve was obtained by selectively removing different fingers of the resonator. Each $|S_{21}|$ curve can be associated to a specific code (as shown in the figure). Figure 8 shows the prototype that was fabricated using the same materials and technique used for the multi-stopband structure.

## 3. Experimental Characterization

The two devices were characterized experimentally through vector network analyzer (VNA) measurements of the $S_{21}$. Measurements were performed by connecting the two ports of the tag to the two ports of the VNA R&S ZVA50, after short-open-load-thru calibration.

**Figure 9:**
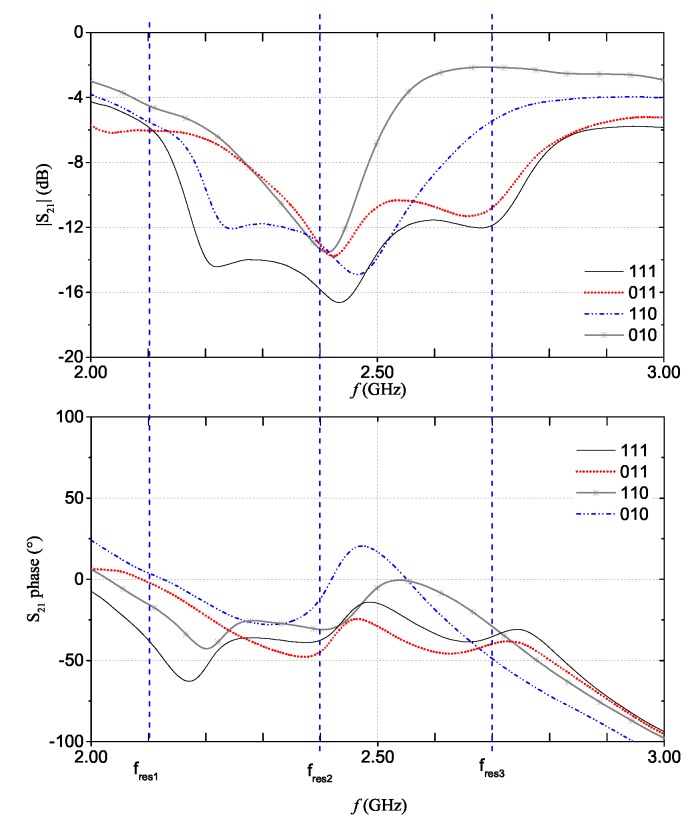
Measurement results for the multi-stopband structure: measurements of the magnitude (**top**) and phase (**bottom**) of the $S_{21}$ for ‘111’, ‘011’, ‘110’, and ‘010’ codes.

**Figure 10:**
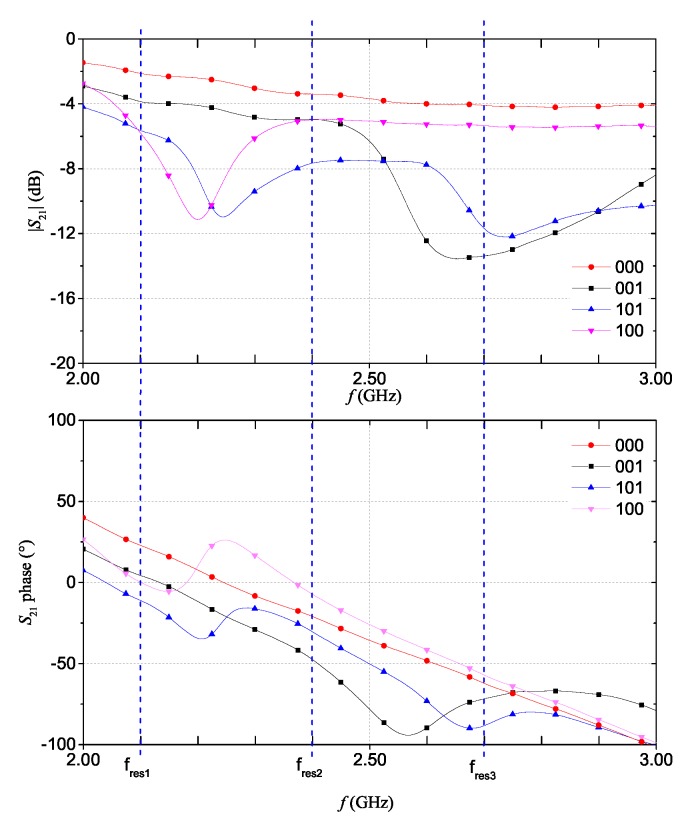
Measurement results for the multi-stopband structure: measurements of the magnitude (**top**) and phase (**bottom**) of the $S_{21}$ for ‘000’, ‘001’, ‘101’, and ‘100’ codes.

For the fully-textile tag relying on resonance-based coding, the $S_{21}$ was measured for all the eight configurations achievable with the three resonators. In practice, the suppression of one resonance (hence, a logic ‘0’) is obtained by short-circuiting the resonator. In particular, a NWCF strip (dashed rectangle in Figure 1c) was used to selectively short-circuit the resonators. Figure 9 and Figure 10 show the measurement results for the magnitude and phase of the $S_{21}$ (results are grouped into different plots only to improve readability). The dashed vertical lines indicate the design resonance frequency values. Experimental results show a general good agreement with the numerical results reported in Figure 4a,b. With regard to the phase, in the ‘111’ configuration, it can be noticed the typical phase variation associated to the three resonances.

Similar considerations apply to all the other seven configurations. The obtained results demonstrate the suitability of this technique for encoding data in chipless wearable devices. However, one limitation is that the number of bits is limited by the number of resonators (and, hence, by size constraints).

**Figure 11:**
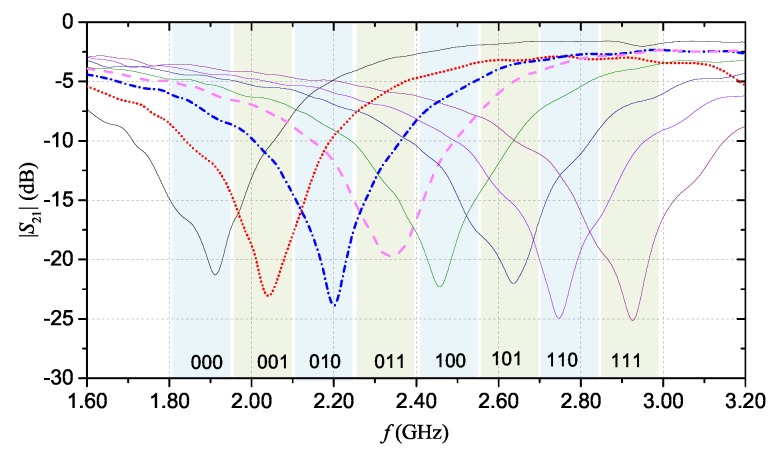
Measurement results of the $|S_{21}|$ for the hairpin resonator.

Also for the hairpin resonator tag, the $S_{21}$ was measured with different configurations of the fingers. Figure 11 shows the measured magnitude of the $S_{21}$. Also for this device, a general good agreement between the experimental results and the simulation results shown in Figure 7 can be observed.

**Figure 12:**
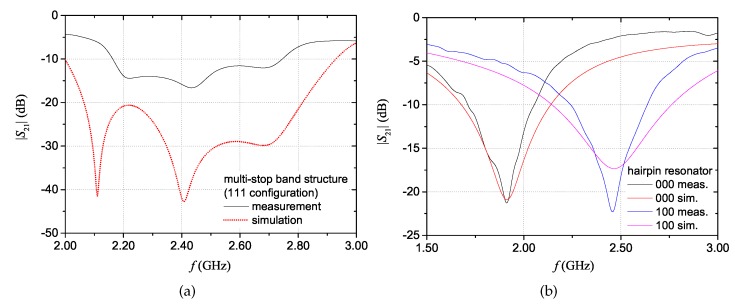
Direct comparison between simulation and measurement results for the multi-stopband tag (**a**) and for the hairpin resonator tag (**b**).

Figure 12a shows the difference between simulation results and measurement results for the multi-stopband structure in the ‘111’ configuration. Differences between the design resonance frequencies and the measured ones (and of the corresponding $|S_{21}|$ amplitudes) are attributable to imperfections of the fabrication process. In particular, the prototype was fabricated on a 1 mm-thick layer of fleece obtained by sewing two 0.5 mm-thick layers. By analysing the prototype, it was observed that the hand-stitching led to a non-uniform thickness of the pile. Additionally, other imperfections were introduced by the cutting plotter used for shaping the conductive parts; in fact, the adopted plotter is more suitable for processing small areas. Another reason of the discrepancy may be attributed to a slight underestimation of the dielectric permittivity of fleece in the design phase.

Figure 12b shows the comparison between measurement and simulation results for the hairpin structure in the configurations corresponding to ‘000’ and to ‘100’. For the hairpin resonator structure, some differences between measurement and simulation results can be attributed to a slight underestimation of the material losses assumed in the full-wave simulations. Overall, frequency-shift coding allows to obtain a more compact size of the device with respect to resonance-based coding.

## 4. Effect of the Environment on the Performance of the Devices

### 4.1. Effect of Bending

**Figure 13:**
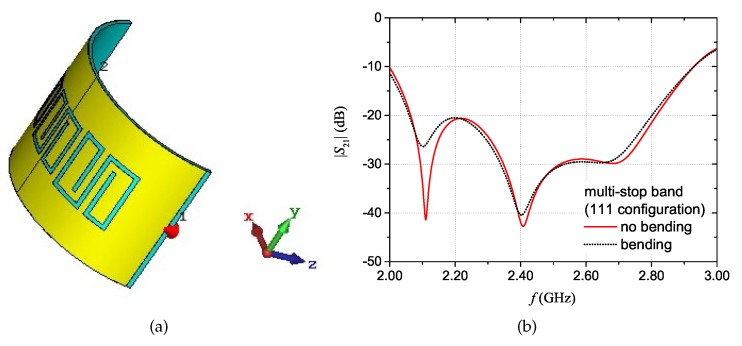
Multi-stopband structure: curved configuration (**a**) and simulation results of the magnitude of the $S_{21}$ with “bending” and with “no bending” of the structure (**b**).

For wearable textile devices, it is important to consider possible variations of the performance caused, for example, by bending. In practical applications, the effect of bending or stretching could be limited by integrating the device on position of clothes which end up covering flat parts of the body (e.g., the upper chest). Another useful strategy is to verify, in the design phase, that the designed structure is robust towards bending. To this purpose, for the proposed prototype configurations, full-wave simulations were carried out considering the curved tag. Figure 13a shows the layout of the simulation for the curved multi-stopband structure, where the radius of curvature was set to 30 mm. Figure 13b shows the comparison between the magnitude of the $S_{21}$ with “bending” and with “no bending”. Results show that there is a variation of the amplitude of the mimima peaks; nonetheless, the resonance frequency values remain approximately the same in the two conditions.

**Figure 14:**
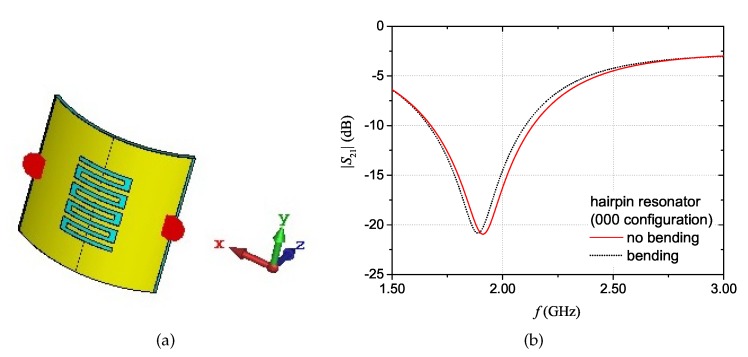
Hairpin resonator structure: curved configuration (**a**) and simulation results of the magnitude of the $S_{21}$ with “bending” and with “no bending” of the structure (**b**).

A similar set of simulations was carried out for the hairpin resonator. The curved configuration layout is shown in Figure 14a. The simulation results for the $|S_{21}|$ with “bending” and with “no bending” are reported in Figure 14b. In this case, it can be noticed that there is a difference of approximately 20 MHz between the resonant frequency values of the two curves. This value, however, is within the limit that could cause ambiguity (i.e. the resolution band discussed in Section 2.3). The reported results suggest that, in practical applications, it is recommended to make sure that the resolution band guarantees unambiguity in the interpretation of results, even in presence of bending.

**Figure 15:**
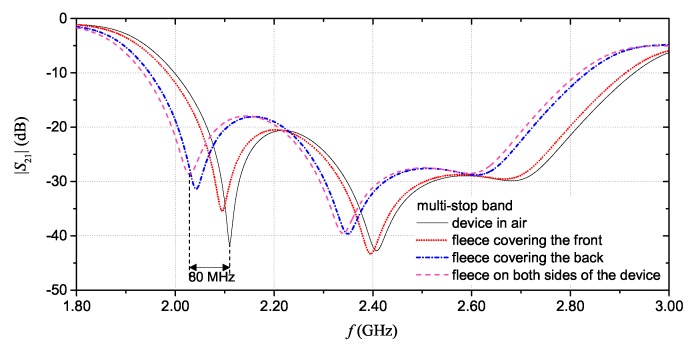
Multi-stopband device: $|S_{21}|$ simulation results when a 5 mm-thick layer of fleece covers the front of the device; the back of the device; and both sides of the device. Results are compared with the $|S_{21}|$ of the device in air (i.e.,with no additional cover).

Finally, when dealing with wearable devices, it is also necessary to assess the performance variation that can be caused for example by the presence of clothing that covers the tag (e.g., a coat). To address this issue, full-wave simulations were carried out considering three cases: 5 mm of fleece covering the front of the device (i.e., covering the microstrip line); 5 mm of fleece covering the back of the device (i.e., covering the resonators); and 5 mm of fleece on each side of the device. Figure 15 shows the obtained results. For the multi-stopband device, results showed that, when both the front and the back of the device are covered with additional clothing, the resonance frequency values of the resonators change of, at worst, 80 MHz. This may cause ambiguity in the association of a resonance peak to a logic “1” or “0”. However, to avoid such an ambiguity, it would suffice to establish a frequency band within which the resonance peak of a resonator must fall to be associated to a logic “1” or “0”. Similar considerations can be applied for the hairpin resonator structure, as mentioned in Section 2.3.

### 4.2. Performance of the Device in Contact with the Body

**Figure 16:**
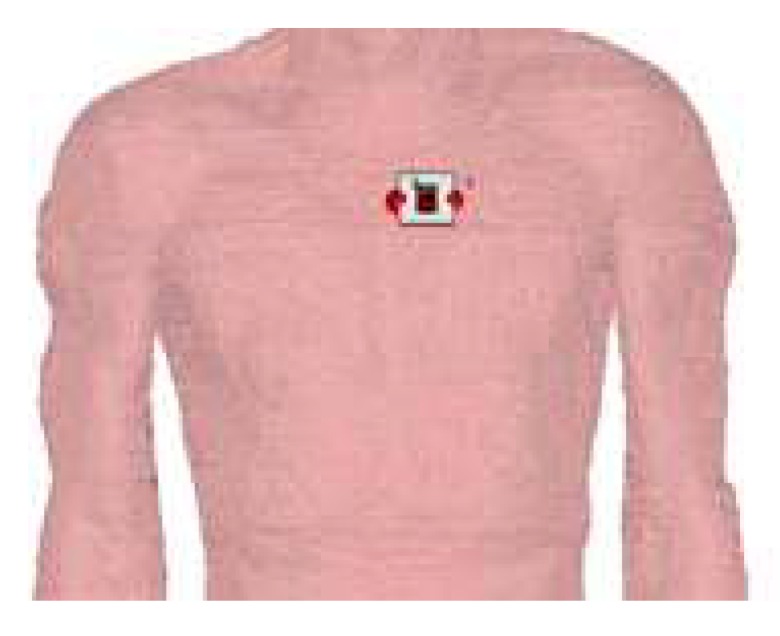
Layout configuration of the simulation of the device in contact with the body.

To assess the performance of the devices in the foreseen practical conditions, simulations were also carried out by considering the device in contact with the human body. For this purpose, additional simulations were carried out using a numerical human phantom, called Duke (developed by IT’IS Foundation). This phantom, which was developed with a resolution of 0.5 mm, is anthropomorphically shaped and loaded with tissue simulating materials. For the simulations, the Duke phantom was imported in CST Microwave Studio. Figure 16 shows the configuration considered in the simulations. The textile device is placed on the chest of the phantom, following the natural curvature of the body. In particular, the front of the device (i.e., the microstrip) is placed in contact with the body, while the resonator faces the air.

**Figure 17:**
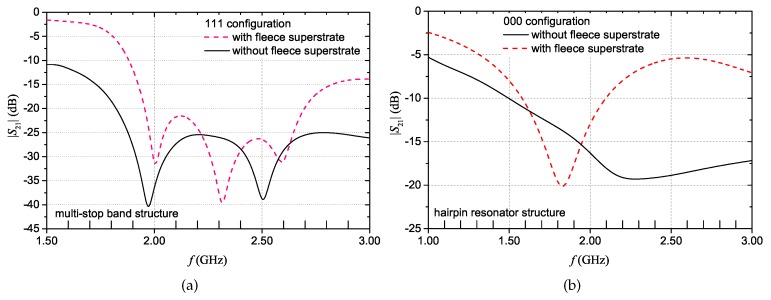
$|S_{21}|$ simulation results obtained placing the devices in contact with the human body: for the multi-stopband structure (**a**) and for the hairpin resonator (**b**), with and without fleece superstate.

Figure 17a shows the $|S_{21}|$ results for the multi-stopband structure in contact with the body (*without fleece superstrate* curve). The simulated configuration is the ‘111’ configuration. It can be noticed that the performance of the device degrades. In particular, the resonance frequency values have shifted towards lower frequencies (as also confirmed by the phase results, not reported for the sake of brevity). Additionally, one resonance peak has completely disappeared. Such a performance degradation is due to the losses introduced by the direct contact of the tag with the body. Hence, to restore the performance of the device, it is necessary to avoid the direct contact with the skin. This can be accomplished, for example, by applying a superstrate of non-conductive material on top of the microstrip line. Using fleece as a superstrate also preserves the textile feature of the structure. The obtained simulation results demonstrate that by employing an additional layer of fleece between the microstrip and the body, the resonant behaviour is restored (*with fleece superstrate* curve of Figure 17a).

The same set of simulations was carried out also for the hairpin resonator structure. In this case, the hairpin configuration corresponding to the ‘000’ code of Figure 7 was simulated. The obtained results are shown in Figure 17b. Also in this case, two simulations were carried out: with and without a layer of fleece placed between the chest and the microstrip line. Once again, the presence of the fleece layer restores the good performance of the structure. As a matter of fact, in view of practical applications, not only would the fleece superstrate restore the resonant behavior, but it would also have the beneficial effect of making more natural the person’s sensation in wearing the textile device.

## 5. A Potential Application in Tracking Leather Products

One of the industrial fields in which these chipless devices may be particularly useful is for the tracing and tracking of upmarket fashion products. The traceability of the supply chain is necessary to guarantee the origin and authenticity of the product. Hence, it becomes crucial to develop innovative and industrializable technological solutions to allow traceability of goods throughout the production chain. In particular, in the manufacturing industry of leather products, tracking the origin of the finished product is still a major issue. This is because of the inherent fragmentation of the related supply chain. Indeed, the tanning process still remains the weakest link in the tracking of hides. This production phase is often outsourced, and hides are fraudulently replaced with poor quality leather. The tanning process completely changes the appearance of the leather obtained compared to raw leather; consequently, when the hides are sent to the tanning and return to the company, it is not possible to verify that the product that was received is the same material that was originally sent. Unfortunately, conventional tracking methods do not withstand the intensive mechanical and chemical processes (such as fleshing, depilation, etc.) of the tanning process. In such applications, the proposed wearable devices could be directly integrated with the hides, in order to resist to the leather manufacturing process. In particular, the leather could be used as a substrate for the chipless tag, while the conductive portions of the chipless device could be sewn on both sides of the leather. Alternatively, the conductive parts of the chipless tag could be made by tattooing conductive materials on the hide, which would make the chipless tag permanently embedded into the leather. Preliminary tests have proven that the tattoo resist the harsh tanning process [33]; however, additional effort should be dedicated to achieving a good uniformity of the thickness of the tattoo and a good geometrical resolution so as to satisfy the high-accuracy geometry required in the fabrication of electromagnetic devices.

## 6. Conclusions

In this work, two fully-textile wearable chipless devices were designed, fabricated and characterized. The combined use of a layer of fleece and of an adhesive non-woven conductive fabric allows to obtain devices that are entirely textile. Additionally, by resorting to suitable encoding techniques (based on the frequency signature of the device), it is possible to accomplish radio-frequency identification, still avoiding the use of electronic chips. Multi-stopband devices are easier to be fabricated and implemented; still, the number of bits is limited by the number of resonators (and, hence, by size constraints). On the other hand, frequency-shift coding allows to obtain a more compact size. However, the results obtained for both the fully-textile prototypes confirm the suitability of these solutions for achieving a seamless and robust integration of wearables with clothes, thus making the proposed strategies particularly useful for a more effective involvement of the user with the IoT sensory environment.

## Figures and Tables

**Table 1:** Dimensions of the multi-stopband structure.

Parameter	Dimension (mm)
$W_{1}$	80.0
$L_{1}$	40.0
$W_{feed1}$	3.7
$W_{res1}$	18.7
$l_{res1}$	18.0
$t_{res1}$	1.2
$d_{res1}$	1.9
$g_{res1}$	2.0

**Table 2:** Dimension of the hairpin resonator.

Parameter	Dimension (mm)
$W_{2}$	40.00
$L_{2}$	40.00
$W_{feed2}$	3.70
$W_{res2}$	14.40
$l_{res2}$	21.60
$t_{fe}$	1.35
$g_{f2}$	1.20
